# Vitamin D nutritional status and vitamin D regulated antimicrobial peptides in serum and pleural fluid of patients with infectious and noninfectious pleural effusions

**DOI:** 10.1186/s12890-016-0259-4

**Published:** 2016-07-08

**Authors:** Carlos A. Amado, María T. García-Unzueta, M. Carmen Fariñas, Francisca Santos, María Ortiz, Pedro Muñoz-Cacho, José A. Amado

**Affiliations:** Division of Pneumology, Universidad de Cantabria, IDIVAL, Santander, Spain; Clinical Biochemistry, Universidad de Cantabria, IDIVAL, Santander, Spain; Infectious Diseases Hospital Universitario Marqués de Valdecilla (HUMV), Universidad de Cantabria, IDIVAL, Santander, Spain; Gerencia Atención Primaria, Servicio Cántabro de Salud, Santander, Spain; Division of Endocrinology, Hospital Universitario Marqués de Valdecilla, Universidad de Cantabria, IDIVAL, Santander, Spain

**Keywords:** Pleural effusion, LL-37, Cathelicidin, β-defensin-2, 25 OH vitamin D

## Abstract

**Background:**

Vitamin D and vitamin D dependent antimicrobial peptides such as Cathelicidin (LL-37) and β-defensin 2 have an important role in innate and adaptative immunity, but their role in pleural effusions has not been studied before.

**Methods:**

Serum and pleural fluid samples from 152 patients with pleural effusion were collected, corresponding to 45 transudates and 107 exudates, 51 infectious effusions (14 complicated and 37 non-complicated), 44 congestive heart failure effusions and 38 malignant effusions. The levels of 25 OH-vitamin D, 1,25-(OH)2-vitamin D, Vitamin D Binding Protein (VDBP), LL-37 and β-defensin 2, both in serum and pleural fluid were evaluated in this prospective study. Differences between groups were analysed using unpaired t tests or Mann–Whitney tests. Correlations between data sets were examined using Pearson correlation coefficient or Spearman rank correlation coefficient. Diagnostic accuracy was estimated using ROC curve analysis.

**Results:**

Low serum 25 OH vitamin D levels were found in all groups. Infectious effusions (IE) had higher serum and pleural fluid LL-37 levels compared to congestive heart failure or malignant effusions. Among IE, complicated had higher serum and pleural fluid LL-37 levels, and lower serum β-defensin-2 levels. Positive correlations were found between serum 25 OH-vitamin D levels and serum or pleural 1,25-(OH)2-vitamin D levels, and between 1,25-(OH)2-vitamin D and LL-37 serum. Diagnostic accuracy of the different molecules was moderate at best.

**Conclusions:**

Hypovitaminosis D is highly prevalent in pleural effusions. LL-37 is produced intrapleurally in IE. This production is higher in complicated IE. No evidence of pleural production of β-defensin 2 was found in any of the groups. Diagnostic accuracy of the different molecules is at the best moderate for discriminating different types of effusions.

## Background

Vitamin D modulates maturation, proliferation and function of different cells of both innate and adaptative immune system [1]. Vitamin D nutritional status is evaluated measuring serum 25 OH vitamin D, the stable metabolite of vitamin D. This molecule is the precursor of calcitriol or 1,25 (OH)2 vitamin D, the active metabolite of vitamin D [2]. Hypovitaminosis D is a highly prevalent situation in hospitalized patients [3]. Multiple studies have shown that vitamin D and its antimicrobial dependent molecules play a defensive role in different infectious diseases such as tuberculosis, bacterial or viral infections [4–6]. Liu et al. [7] demonstrated that some *M. tuberculosis* antigens were recognized by Toll-Like 2/1 receptors of monocytes and innate immunity cells (skin, respiratory, digestive and urinary epithelium, etc.). This was followed by an intracrine activation of 1α-hydroxylase, synthesis of 1,25 (OH)2 vitamin D, and activation of the vitamin D receptor, inducing the synthesis and release of LL-37 (also known as human cathelicidin) and β-defensin-2. These molecules have important antimicrobial effects against virus, bacteria, and fungus in the lung [5, 6]. On the other hand, Vitamin D-binding protein (VDBP) is a multifunctional serum protein that binds vitamin D and its metabolites and transports them to target tissues [8]. VDBP is also a precursor of a macrophage activating factor [8].

There are no data in the literature regarding the prevalence of hypovitaminosis D in pleural effusions. An old study by Barnes et al. [9] showed that pleural levels of calcitriol were significantly higher in tuberculous pleural effusions as compared with cardiac effusions, although the number of patients studied was very small. On the other hand, in tuberculous patients pleural levels of 1,25 (OH)2 vitamin D were significantly higher than serum levels. These data suggested intrapleural synthesis of 1,25 (OH)2 vitamin D in tuberculosis, followed by release to blood. Otherwise, these aspects have never been studied in non-tuberculous pleural effusions.

One study measured VDBP in pleural fluid and serum [10]. Pleural fluid VDBP and VDBP pleural fluid/serum ratio were significantly higher in bacterial effusions compared to tuberculous or malignant. However, no differences were found in the serum VDBP/total protein ratio.

The possible defensive role of LL-37 in infectious pleural effusions has never been studied before. On the other hand, it is known that β-defensin-2 can be produced in vitro by pleural mesothelial cells when stimulated by *Staphylococcus* peptidoglycan [11]. This interesting finding suggests that these cells may have an important role in innate immunity of the pleural cavity, but there are no data in vivo in humans.

In this study we aimed to measure serum and pleural fluid levels of 25 OH vitamin D, 1,25 (OH)2 vitamin D, VDBP, LL-37 and β-defensin-2 in patients with different etiological types of pleural effusions. We hypothesized that infectious effusions should have higher levels of serum or pleural calcitriol, LL-37 and β-defensin-2, reflecting a higher inflammatory response.

## Methods

### Study subjects

**Fig. 1:**
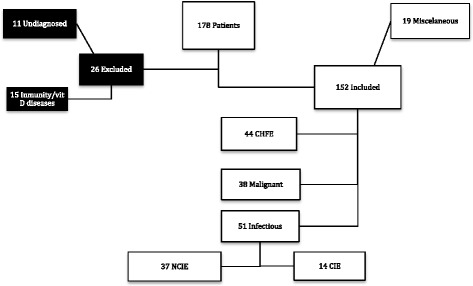
Flow Chart: Vit D = vitamin D; CHFE = Congestive Heart Failure Effusion; NCIE = Non-complicated effusion; CIE = complicated effusion

This is a prospective study approved by the Ethics Committee of Cantabria (CEIC Cantabria 25/2012). All patients gave informed consent to obtain samples of blood and pleural fluid at diagnosis time. We collected pleural fluid and serum at time of diagnosis of patients who were admitted in our hospital with the clinical diagnosis of pleural effusion along a period of 18 months. Patients with diseases or drugs known to alter vitamin D metabolism or immunological responses and patients with pleural effusion of unknown ethiology were excluded. In this way, one hundred and fifty two patients managed by the same medical team were recruited as explained in Fig. 1.

### Classification

Patients were diagnosed of: 1) Congestive heart failure effusion (CHFE), *n* = 44, based on clinical grounds, according to the AHA guidelines, 2) Malignant effusion (ME), *n* = 38, when malignant cells were found in pleural effusion or pleural biopsy, 3) Infectious effusion (IE), *n* = 51, when clinical findings were compatible with pneumonia and there was response to antimicrobial treatment and/or positive culture results. There was also a miscellaneous group of 19 patients with pleural effusion due to other less frequent causes (pulmonary thromboembolism *n* = 7, pancreatitis *n* = 6, postoperative *n* = 5, and nefrotic syndrome *n* = 1, that was not separately analyzed. Pleural fluids were also classified as exudates *n* = 107 or transudates *n* = 45, according to the cause of the effusion in consonance with Light criteria [12]. Patients with infectious effusions who received chest tubes for the treatment were classified as having complicated effusions (CIE). The decision to place a chest tube was left to the discretion of the attending physician, although it is likely that this decision was at least partially influenced by the biochemical characteristics of the fluid. Finally, we studied 37 noncomplicated (NCIE) and 14 CIE.

### Biochemical analyses

Serum and pleural effusion samples were collected in the appropriate way for the different assays. Routine determinations were done using the standard methods of our Hospital. Serum and pleural fluid aliquots for this specific study were stored at −80 °C until assayed.

25 OH vitamin D levels were measured by the automated chemiluminiscence competitive assay IDS iSYS (Immunodiagnostic Systems Ltd, Boldon, UK), as previously reported [13]. Our laboratory is DEQAS (Vitamin D External Quality Assessment Scheme) certified. We considered hipovitaminosis D when serum levels of 25 OH vitamin D were lower than 20 ng/ml, according to the 2011 Food and Nutrition Board dietary references intakes for vitamin D and calcium. 1,25(OH)2 vitamin D levels were measured by radioimmunoassay (DiaSorin Inc, Stillwater MN USA). VDBP, LL-37 and β-defensin-2 levels were measured by ELISA using the following kits: Quantikine Human Vitamin D Binding Protein (R&D Systems Europe, Abingdon, UK), HK321 Human LL-37 (Hycult Biotech, Uden, Holland) and human β-defensin-2 (Alpha Diagnostic International, San Antonio, TX USA).

### Statistical analysis

Data are presented as mean ± SD values for normally distributed data or median (interquartile range, IQR) for nonparametric data. Differences between proportions were calculated using chi-square test. Differences between groups were analyzed using unpaired t tests or Mann–Whitney tests. Correlations between data sets were examined using Pearson (r) correlation coefficient or Spearman rank (rs) correlation coefficient. Differences were considered significant for all statistical tests at *p* values of less than 0.05. All reported p values are two-sided. Analysis was performed using statistical computer Software (IBM SPSS Statistics) version 20.00 for Windows. ROC curves analysis was performed using software MEDCALC version 11.6.1.0 (MedCalc Software, Mariakerke, Belgium). The database and leyend of the database supporting the conclusions of this article are included within its additional files.

## Results

Tables 1 and 2 show the principal results.

**Table 1:** Biochemical Parameters in Pleural effusion

Parameter		IE	ME	CHFE	*P* Value	Exudate	Transudate	*P* Value	CIE	NCIE	*P* Value
WBC (cells/μL)	Serum	10715 ± 5088	9435 ± 3451	9453 ± 5403	0.32	10156 ± 4575	9278 ± 5456	0.368	10969 ± 4457	10609 ± 5396	0.834
	Pleural	555 (160, 960)	400 (240, 680)	240 (160, 640)	o.149	410 (240, 800)	240 (160, 640)	0.046	560 (160, 800)	550 (160, 960)	0.761
N, (%)	Serum	74 (68, 80)	71 (61, 76.7)	75 (66, 81)	0.102	74 (68, 80)	74 (62, 81)	0.55	71.5 (68, 80)	74 (65, 81)	0.718
	Pleural	50 (17.5, 72.5)	15 (10, 20)	15 (5, 25)	0.001	20 (10, 55)	15 (5, 25)	0.026	58 ± 24	40 ± 29	0.111
pH	Pleural	7.23 ± 0.31	7.37 ± 0.07	7.4 ± 0.11	0.03	7.28 ± 0.26	7.4 ± 0.11	0.016	7.03 ± 0.32	7.30 ± 0.29	0.028
Glucose (mg/dL)	Pleural	99,47 ± 51.95	109 ± 42	133.67 ± 45.4	0.01	105.05 ± 50.98	133.67 ± 45.43	0.07	78.00 ± 58	107.68 ± 47.61	0.080
LDH (IU/L)	Pleural	270 (140, 735)	329 (144, 436)	123 (88, 270)	0.001	283 (134, 528)	119 (84, 261)	0.001	682 (164, 1771)	243 (121, 414)	0.035

*IE* Infectious effusions, *CHFE* Congestive heart failure effusions, *ME* Malignant effusions, *CIE* Complicated infectious effusions, *NCIE* Non complicated infectious effusions, *WBC* White blood cells, *N* Neutrophils, *LDH* Lactate Dehydrogenase. Data presented as mean ± SD for parametric results and mean (interquartilic range)

**Table 2:** Vitamin D related molecules in Pleural effusion

Parameter		IE	ME	CHFE	*P* Value	Exudate	Transud.	*P* Value	CIE	NCIE	*P* Value
25OH Vitamin D (ng/mL)	Serum	12 (5, 84)	18.4 (6.9, 41)	10,4 (3, 39.6)	0,001	12.8 (5, 84)	10.6 (3, 39,6)	0,019	11.85 (9.2, 16.2)	13.5 (7.6, 22.4)	0.731
	Pleura	16 (1, 63)	17 (5.5, 47)	12.2 (1, 30.5	0,016	15 (1, 63)	12 (1, 25)	0,048	16 (9.65, 20.65)	15 (7.75, 25.32)	0.551
1,25 (OH)2 Vitamin D (pg/mL)	Serum	25.65 (7, 113)	46 (9, 140)	22.5 (5, 116)	0,043	25.65 (5, 140)	22 (8, 66)	0,708	22 (14.5, 35.5)	32 (8.5, 41.5)	0.751
	Pleura	22 (4, 132)	31.5 (0.4, 1.28)	12 (2, 52)	0,001	18 (4, 132)	12 (2, 50)	0,030	22 (12, 34)	21.5 (7.2, 32.5)	0.627
VDBP (mg/L)	Serum	203 (29, 494)	275.10 (13.13, 730)	197 (27, 1122)	0,1	236 (13, 730	190 (27, 1122)	0,307	139 (29, 285)	166 (42, 338)	0.71
	Pleura	134 (4, 834)	191 (5, 464)	95 (10, 446)	0,001	155 (4, 834)	95 (10, 446)	0,48	85 (10, 183)	217 (30, 258)	0.112
LL-37 (ng/mL)	Serum	0.84 (0.01, 153)	0.47 (0.01, 12.62)	0.25 (0.01, 10.34)	0.017	0.67 (0.01, 153)	0,.24 (0.01, 10.3)	0.017	0,51 (0.28, 1.29)	1.43 (0.95, 1.85)	0.029
	Pleura	1.42 (0.05, 153)	0.52 (0.01, 13.51)	0.3 (0.01, 3.3)	0.001	0.91 (0.01, 153)	0.3 (0.01, 3.3)	0..001	1.05 (0.47, 4.28)	3.11 (1,62, 5.62)	0.014
β-defensin-2 (pg/mL)	Serum	232 (14, 2100)	224 (10, 3000)	284 (9, 1834	0.966	213 (9, 3000)	258 (9, 1834)	0.651	431 (136, 1015)	112 (71, 233)	0.023
	Pleura	265.2 (6, 1293)	276 (25, 3000)	241 (5, 2206)	0.706	240 (6, 3000)	214 (5, 2206)	0.484	383 (72, 804)	152 (16, 352)	0.091

*IE* Infectious effusions, *CHFE* Congestive heart failure effusions, *ME* Malignant effusions, *CIE* Complicated infectious effusions, *NCIE* Non complicated infectious effusions, *WBC* White blood cells, *N* Neutrophils, *LDH* Lactate Dehydrogenase, *VDBP* Vitamin D Binding Protein, *LL-37* Leucin leucin 37 (Cathelicidin). Data presented as mean ± SD for parametric results and mean (interquartilic range)

### Blood and pleural fluid cytologic and biochemical parameters

As expected, IE had higher levels of neutrophils and Lactate dehydrogenase (LDH), and lower pH and glucose levels in the pleural fluid compared to CHFE. Exudates had higher levels of pleural fluid leukocytes, neutrophils and LDH and lower glucose and pH levels than transudates. CIE had a higher level of LDH and a lower pH than the NCIE.

### Serum and pleural fluid vitamin D and vitamin D related molecules

There were no seasonal differences in the dates of obtaining the samples of the different groups (*p* = 0.102).

34 patients (77 %) in the CHFE group, 33 (64 %) in the IE group and 20 (52 %) in the malignant group had vitamin D deficiency (*p* < 0.001). There were 35 (77 %) patients with transudative effusion presenting serum 25 OH levels below 20 ng/ml and 75 (65 %) in the exudative group (*p* = NS). There were 10 patients with 25 OH levels below 20 ng/ml in the CIE group (71 %) and 23 (62 %) in the NCIE group (*p* = NS).

ME had higher levels of serum 25OH vitamin D compared to CHFE and IE (*p* < 0.001 and *p* = 0.01). Similar differences were found in 1,25 (OH)2 vitamin D levels (*p* = 0.041 and *p* = 0.019). Exudative effusions had higher levels of 25 OH vitamin D compared to transudative effusions (*p* = 0,019). Pleural fluid levels of 1,25 (OH)2 vitamin D were lower in CHFE compared with infectious or malignant effusions (*p* = 0.033 and *p* < 0.001). No significant difference was found in serum or pleural fluid levels of 25 OH vitamin D, 1,25 (OH)2 vitamin D or VDBP between CIE and NCIE.

IE had higher levels of serum cathelicidin compared to CHFPE or ME (*p* = 0.005 and *p* = 0.017). Pleural levels of LL 37 were also higher in the IE group (*p* = 0.001 and *p* = 0.001). Exudative effusions had higher serum levels of LL-37 compared to transudative effusions (*p* = 0.017). CIE had significantly higher levels of LL-37, both in serum (*p* = 0.029) and pleural fluid (*p* = 0.014) compared with the NCIE group. No differences in serum or pleural β-defensin-2 levels were found among the different etiological groups or between exudate and transudate groups. In contrast, β-defensin-2 serum levels were lower in CIE (*p* = 0.023); otherwise, a non-significant trend for lower levels was found in CIE pleural fluid compared with the NCIE group (*p* = 0.091).

### Relationships among vitamin D related molecules

When all patients were considered, significant positive correlations were found between serum and pleural levels of 25 OH Vitamin D (rs = 0.596, *p* < 0.001), 1,25 (OH)2 vitamin D (rs = 0.516, *p* = 0.001), VDBP (rs = 0.870, *p* < 0.001), LL-37 (rs = 0.707, *p* < 0.001) and β-defensin-2 (rs = 0.870, *p* < 0.001).

Interestingly, serum levels of 25 OH vitamin D also showed significant positive correlations with pleural (rs = 0.54, *p* < 0.001) 1,25 (OH)2 vitamin D levels. Furthermore, serum 1,25 (OH)2 vitamin D levels were significantly correlated with serum LL-37 levels (rs = 0.388, *p* = 0.05), but not with serum β-defensin-2 levels.

On the other hand, vitamin D metabolites and related molecules did not correlate with glucose, pH, or LDH, either in serum or in pleural effusion.

### Diagnostic accuracy of vitamin D related molecules

Evaluation of ROC plots in serum and pleural fluid samples taken from patients with effusions for the discrimination between infectious origin and other origin were similar for pleural fluid LL-37 [AUC = 0.732 (95 % CI, 0.657-0.798), *p* < 0.001] and pleural fluid 1,25 (OH)2 vitamin D [AUC = 0.754 (95 % CI, 0.654-0.838), *p* < 0.001].

Pleural fluid LL-37 [AUC = 0.657 (95 % CI, 0.575-0.733), *p* = 0,0016] was superior to serum LL-37 [AUC = 0.586 (95 % CI, 0.503-0.667), *p* = 0,1181] for the differentiation between exudative and transudative effusions.

Pleural fluid LL-37 [AUC = 0.731 (95 % CI, 0.585-0.847), *p* = 0.0012] and serum β-defensin2 [AUC = 0.731 (95 % CI, 0.562-0.861), *p* = 0.0085] were superior to serum LL-37 [AUC = 0.708 (95 % CI, 0.557-0.831), *p* = 0.0015] for the differentiation between CIE and NCIE.

## Discussion

In 1998 Thomas et al. [3] showed in an influential paper that the prevalence of hypovitaminosis D was high in non-selected inpatient subjects from the medical wards of the Massachusetts General Hospital in Boston. A multivariant study demonstrated different factors related to hypovitaminosis, such as advanced age, insufficient ingestion of vitamin D or sun exposure, presence of specific diseases (nephrotic syndrome, malabsorption, hepatic cirrhosis, morbid obesity etc.) and use of drugs that increase vitamin D catabolism. In that study no patients with pleural effusion were included, and since then the prevalence of hypovitaminosis D in pleural effusions has not been specifically studied. Our study shows, as expected, that all kinds of pleural effusion are associated frequently with hypovitaminosis D defined as 25 OH vitamin D serum levels lower than 20 ng/ml.

Significant lower levels of serum 1,25 (OH)2 vitamin D were found in CHFE. This might probably be due to lower levels of 25 OH vitamin D in this group. This is supported by the positive correlation between 25 OH vitamin D and 1,25 (OH)2 vitamin D levels. This correlation is typical of hypovitaminosis D, and it means that the substrate (25 OH vitamin D) is really a limiting factor for the production of 1,25 (OH)2 vitamin D. If 25 OH vitamin D levels were normal (plenty of substrate) this correlation should not be found. No significant differences were found in either serum or pleural fluid levels of 25 OH vitamin D, 1,25 (OH)2 vitamin D or VDBP in CIE vs. NCIE. We had hypothesized that pleural 1,25 (OH)2 vitamin D could be increased in complicated patients, but our data does not support this hypothesis. This can be due, at least in part, to the fact that low levels of the precursor molecule 25 OH vitamin D limit the production of 1,25 (OH)2 vitamin D. Furthermore, the paracrine/intracrine production of calcitriol in response to the infection, may not be enough to induce measurable changes in pleural fluid or serum.

On the other hand, we found higher levels of LL-37 both in serum and pleural fluid in IE, specially in CIE, suggesting that, despite hypovitaminosis D, innate immune cells are able to produce higher quantities of this natural antimicrobial molecule when they are severely challenged. The positive correlation between serum 1,25 (OH)2 vitamin D and LL-37 suggests that higher levels of the active metabolite in fact potentiate LL-37 synthesis, even in the presence of hypovitaminosis D. On the contrary serum β-defensin-2 levels were decreased in CIE and did not correlate with 1,25 (OH)_2_ levels. The different behavior of these antibiotic molecules suggests that, although both are modulated by vitamin D, other factors are also importantly involved in their response. For example, it has been demonstrated in vitro that β-defensin-2 responses to vitamin D are lower than those of LL-37 [7]. In vivo, quantity and time course of response of these molecules to different challenges are also different [14, 15]. Furthermore it has been reported that β-defensin-2 decreases in sepsis [16] and lower plasma level of this substance is an independent predictor of adverse outcomes in patients with community-acquired pneumonia [17]. Further time course studies are needed to analyze whether this is due to consumption of β-defensin-2, or because certain patients have lower response of this molecule leading to susceptibility to sepsis.

Significant positive correlations were found between serum and pleural levels of all the molecules studied, suggesting there is good diffusion of these molecules through the pleural membranes. Otherwise, no correlations were found with other established pleural biomarkers, such as pH, glucose or LDH.

Diagnostic accuracy of the different molecules evaluated by ROC analysis is similar or lower to standardized methods used in clinical practice. These results suggest that although some vitamin D related molecules could be markers with relatively good sensitivity and specificity for pleural effusion evaluation, the cost and time needed do not support its use as a routine laboratory test.

Our study has limitations. The number of patients studied is relatively small in some of the groups, especially in the CIE group, and we have only one determination, at the time of diagnosis. Subsequent samples should give a clearer picture of the responses and interactions among all molecules. Likewise, the definition of CIE and NCIE is never clear-cut. In the present study CIE were defined as those that received chest tubes. This decision was made with the knowledge of the pleural fluid pH, glucose and LDH, and this could have influenced which patients were given chest tubes.

## Conclusion

In conclusion hypovitaminosis D is highly prevalent in all kinds of pleural effusions. LL-37 is higher both in pleural fluid and blood of the more inflammatory effusions, but there is a weaker response of β-defensin-2 in blood. The pathophysiological significance of these findings merits to be studied in further detail. Also, a clinical trial aimed at improving the vitamin D nutritional status should test if vitamin D boosts the innate antibiotic and clinical response in these patients.

## Abbreviations

VDBP Vitamin D-binding protein; LL-37 human cathelicidin; CHFE Congestive heart failure effusion; ME Malignant effusion; IE Infectious effusion; CIE complicated effusions; NCIE noncomplicated effusions; DEQAS Vitamin D External Quality Assessment Scheme; LDH Lactate dehydrogenase
